# The Impact of the CALLY Index on All‐Cause Mortality in Patients With Depression: A Longitudinal Analysis Using NHANES Data

**DOI:** 10.1002/hsr2.72387

**Published:** 2026-04-19

**Authors:** Yin Zhang, Rula Sa, Huan Jia, Xusheng Wang, Ruiting Ma, Lijun Tong

**Affiliations:** ^1^ Mental Health College Inner Mongolia Medical University Hohhot China; ^2^ Inner Mongolia Mental Health Center, Inner Mongolia Autonomous Region Hohhot China; ^3^ Inner Mongolia Medical University Hohhot China

**Keywords:** All‐cause mortality, CALLY index, depression, NHANES

## Abstract

**Background and Aim:**

The C‐reactive protein‐albumin‐lymphocyte (CALLY) index is an emerging biomarker, yet, its potential association with all‐cause mortality risk in patients with depression remains unexplored. The present investigation sought to evaluate this potential correlation.

**Methods:**

This analysis included 4,426 adults with depression from the National Health and Nutrition Examination Survey (NHANES) (2005–2010, 2015–2016). The CALLY index was calculated as (lymphocyte count × albumin)/CRP × 10³. Multivariable Cox regression was used to assess its association with all‐cause mortality. Restricted cubic spline (RCS) and Kaplan‐Meier curves examined nonlinear relationships and survival probabilities across strata.

**Results:**

Over a median follow‐up of 121 months, the cumulative all‐cause mortality rate was 14.84%. Cox regression showed that each one‐unit increase in log‐transformed CALLY index was associated with a 41% lower risk of all‐cause mortality. When categorized into quartiles, the highest quartile (Q4) demonstrated significantly lower hazard ratios (HR) across all models compared to the lowest (Q1). Kaplan‐Meier analysis revealed an inverse relationship between CALLY index levels and mortality (*p* < 0.0001). RCS analysis identified a nonlinear l‐shaped association (*p*‐trend < 0.001), with an inflection point at Log‐CALLY = 4.09. Subgroup analyses indicated that smoking status, poverty income ratio(PIR), chronic respiratory diseases(CRD), and diabetes significantly modified this association (all *p* for interaction < 0.05).

**Conclusion:**

The CALLY index was independently associated with all‐cause mortality in patients with depression, with lower values indicating higher long‐term mortality risk. It may serve as a potential prognostic marker reflecting inflammation and nutritional status in this population.

**Abbreviations**
95% CI: 95% Confidence Intervals
BMI: body mass index
CALLY index: C‐reactive protein‐albumin‐lymphocyte index
CHD: coronary heart disease
CHF: congestive heart failure
CRD: chronic respiratory disease
CRP: C‐reactive protein
CVDs: cardiovascular diseases
HR: hazard ratio
IQR: interquartile range
KM: Kaplan‐Meier
MLR: Monocyte‐to‐Lymphocyte Ratio
NCHS: National Center for Health Statistics
NDI: National Death Index
NHANES: National Health and Nutrition Examination Survey
NLR: Neutrophil‐to‐Lymphocyte Ratio
PHQ‐9: Patient Health Questionnaire‐9
PIR: poverty income ratio
PLR: Platelet‐to‐Lymphocyte Ratio
PNI: Prognostic Nutritional Index
RCS: restricted cubic spline

## 1 Introduction

Depression stands as a pervasive and intensifying mental health crisis worldwide [1]. Research reveals that individuals suffering from depression are at nearly twice the risk of developing conditions like diabetes and heart failure compared to the general populace [2, 3]. Moreover, they face a significantly increased risk of premature mortality due to various causes, including cardiovascular diseases and suicide [4, 5]. However, the day‐to‐day management of depressive patients often focuses primarily on changes in psychological state, while related physiological changes have not received adequate attention. Therefore, identifying a biomarker through routine clinical tests to assess the survival risk and prognostic status of patients with depression could help identify potential mortality risks, enable timely interventions, and improve the overall management of these patients.

In the pathophysiological cascade of depression, chronic inflammation, immune dysfunction, and nutritional status are considered key influencing factors. A marker of chronic inflammation, C‐reactive protein (CRP), has garnered attention, with research indicating that over 29% of depressed patients show elevated CRP levels [6]. Elevated baseline C‐reactive protein concentrations in these individuals demonstrate a strong association with greater likelihood of experiencing severe cardiovascular complications, including acute coronary syndromes, cerebrovascular accidents, and fatal cardiac outcomes [7]. Conversely, diminished CRP concentrations correspond with attenuated disease progression and improved clinical outcomes [8]. In addition to inflammation, research has shown that depression is closely related to immune function [9, 10]. Accumulating evidence suggests that dysregulation of immune function—particularly involving lymphocyte dysfunction—contributes substantively to the pathophysiology of major depressive disorder [11]. As a reliable biomarker of nutritional status in chronically ill populations, serum albumin concentrations have emerged as a significant determinant of depressive vulnerability [12, 13]. Specifically, hypoalbuminemia independently predicts increased susceptibility to depression and correlates inversely with symptom severity [14, 15, 16].

The CALLY index, a novel composite parameter, provides a comprehensive evaluation of systemic inflammation and immune capacity by considering CRP, albumin, and lymphocytes [17, 18]. This index has proven predictive in cardiovascular diseases and malignancies. Research involving patients diagnosed with esophageal and colorectal cancers has identified an association between reduced CALLY index and more advanced stages of the disease, as well as diminished survival outcomes [19, 20]. Beyond these specific conditions, the prognostic value of the CALLY index has been extended to complex systemic disorders. For instance, it shows significant prognostic potential in hematological malignancies such as primary myelofibrosis (PMF), particularly in patients with thrombosis history [21]. Large cohort studies further confirm its protective role against cancer risk and survival in lymphomas, highlighting its utility as a versatile marker of systemic inflammation, nutrition, and immunity [22]. A systematic analysis by Zhang [23] and Han [24] confirmed that lower CALLY index values correlate with higher risks of all‐cause and cardiovascular mortality in rheumatoid arthritis patients suffering from cardiovascular complications. Recent studies have provided further evidence of a substantial inverse relationship between the CALLY index and various risks associated with conditions such as diabetes complicated by depression, chronic obstructive pulmonary disease, angina and peripheral artery disease [25, 26, 27, 28]. Although previous research has provided valuable understanding across this spectrum of inflammatory conditions, the relationship between the CALLY index and total mortality rates among depressed populations—a condition intrinsically linked to systemic inflammation remains unexplored. Our research seeks to bridge this knowledge gap by examining the association of the CALLY index with mortality from all causes in depression patients using a longitudinal cohort approach. This investigation will generate robust laboratory‐based data for evaluating mortality risk in this clinically susceptible group.

## 2 Methods

### 2.1 Study Population

**Figure 1:**
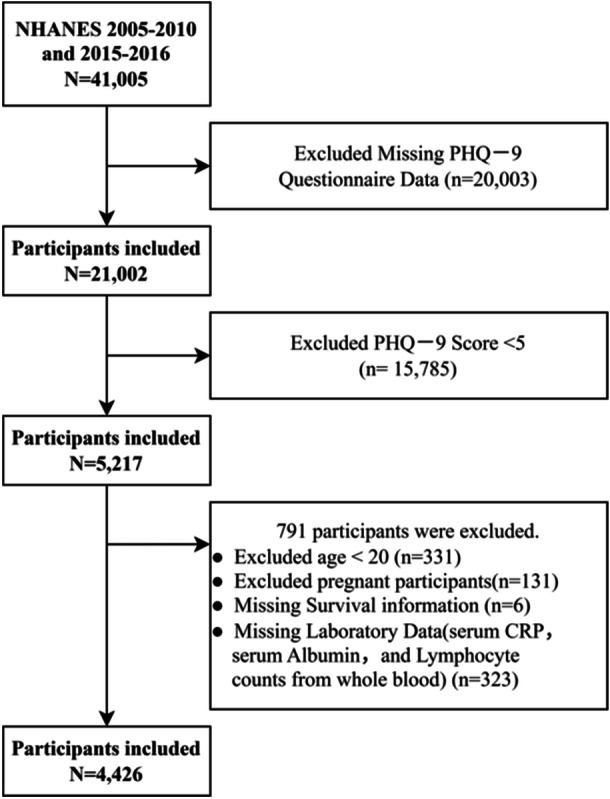
Flowchart for screening participants in the NHANES database.

This study utilized data from the National Health and Nutrition Examination Survey (NHANES) as its population source and methodological foundation. All procedures adhered strictly to the ethical guidelines outlined in the Declaration of Helsinki. All research protocols were approved by the Research Ethics Review Committee of the National Center for Health Statistics. Prior to enrollment, written informed consent was obtained from every participant. Comprehensive details regarding the survey design and data acquisition protocols are available at the official NHANES website: https://wwwn.cdc.gov/nchs/nhanes/Default.aspx. The information utilized in this study was obtained from NHANES survey cycles conducted between 2005 and 2010, as well as from those carried out in 2015 and 2016. The criteria for excluding participants included the following: (1) missing PHQ‐9 questionnaire information (*n* = 20,003), (2) PHQ‐9 score less than 5 (*n* = 15,785), (3) age under 20 years (*n* = 331), (4) pregnancy (*n* = 131), (5) missing survival information (*n* = 6), (6) missing laboratory data (complete blood lymphocyte count, serum albumin and serum CRP (*n* = 323). In conclusion, the final analysis encompassed a total of 4,426 participants (Figure 1).

### 2.2 Depression Evaluation

The assessment of depression was conducted using the Patient Health Questionnaire‐9, a reliable screening instrument comprising nine items. Each item is scored from 0 to 3, corresponding to responses that range from “Not at all” to “Nearly every day.” A cumulative score of 5 or higher indicates the existence of depressive symptoms [29].

### 2.3 Assessment of CALLY Index

The CALLY index is calculated through a mathematical expression involving three key hematological parameters: serum albumin levels (measured in grams per deciliter), absolute lymphocyte count (quantified as cells per microliter), and serum C‐reactive protein concentration (expressed in milligrams per deciliter). This composite biomarker is derived by first multiplying the albumin concentration with the lymphocyte count, then dividing this product by the CRP value, and finally scaling the result by a factor of 10³ to obtain the final index value [17, 18]. Venous specimens were obtained from study participants at an on‐site collection facility and thereafter transported to a reference laboratory for quantitative analysis of C‐reactive protein, serum albumin, and absolute lymphocyte counts using established laboratory procedures. The levels of serum C‐reactive protein were assessed through the latex‐enhanced turbidimetric technique, utilizing both the Behring turbidimeter and the Beckman Coulter Synchron analyzer for accurate measurement [30]. The determination of albumin levels was conducted utilizing the Roche Cobas 6000 analyzer [31].

In the analysis, both continuous and categorical models were implemented. To address the skewness of the CALLY index distribution, a natural logarithmic transformation was performed. Within the categorical model, the log‐CALLY index was categorized into quartiles, using the first quartile (Q1) as the reference group.

### 2.4 Study Population Mortality Rate

The main outcome measured was overall mortality among individuals diagnosed with depression. Data on survival was obtained from the National Death Index (NDI) maintained by the CDC (https://www.cdc.gov/nchs/ndi/index.html). The follow‐up period for each participant was determined from the time of enrollment until either death or the most recent update from the NDI (December 31, 2019).

### 2.5 Covariates

The selection of covariates was guided by established clinical knowledge of depressive disorders and mortality risk factors, incorporating additional variables that could potentially influence the study outcomes. These included demographic characteristics, familial behaviors, physical examinations, biochemical assessments, and pertinent variables from the patients' medical histories. Demographic data included: age (≥ 20 years), sex (female, male), race (Mexican American, other Hispanic, non‐Hispanic White, non‐Hispanic Black, other racial groups), marital status (never married, married/cohabitating, widowed/divorced/separated), education level categorized into three groups: (less than high school, high school or equivalent, college or above), and poverty income ratio (PIR, < 1.30, 1.30–3.50, ≥ 3.50). The assessment of familial behavioral factors incorporated smoking status, characterized as having consumed over 100 cigarettes during one's lifetime, and patterns of alcohol consumption(consuming at least 12 alcoholic beverages annually). Moderate recreational activities (indicated by a response of ‘Yes’ in the survey). Physical examination involved the measurement of body mass index (BMI, kg/m²). Biochemical assessments included:serum albumin, blood lymphocytes and C‐reactive protein. Medical history variables included: Hypertension (self‐reported history of high blood pressure), Diabetes (self‐reported doctor's diagnosis of diabetes) and Cancer (self‐reported doctor's cancer or malignancy), Cardiovascular diseases (CVDs) include coronary heart disease (CHD), angina pectoris, congestive heart failure (CHF), stroke and myocardial infarction, as diagnosed by a healthcare professional. Chronic respiratory diseases (CRD) encompass asthma, emphysema, and chronic bronchitis, as self‐reported by participants answering “Yes” to the corresponding questions.

### 2.6 Statistical Analysis

For quantitative measures that exhibited a normal distribution, data were summarized using means accompanied by standard deviations. Conversely, non‐normally distributed metrics were summarized by their median values alongside the interquartile range (IQR). Statistical analysis of categorical data was performed by calculating frequency distributions and their respective percentage representations. The CALLY index, known for its left‐skewed distribution, underwent logarithmic transformation and was analyzed by quartiles. The study employed a dual analytical approach, evaluating all parameters as both continuous and categorical variables. For continuous data analysis, one‐way ANOVA was implemented, while categorical variables were examined through chi‐square testing. This methodological design allowed for comprehensive assessment of variable characteristics across different measurement scales.

To examine the association between the CALLY index and all‐cause mortality, variables demonstrating statistical significance (*p* < 0.05) in initial univariate analyses were subsequently included in a multivariate Cox proportional hazards regression model [32]. To investigate possible nonlinear associations, restricted cubic splines (RCS) [33] along with a piecewise linear regression approach were utilized, while also evaluate the threshold effect of the CALLY index on mortality. We conducted an exploratory assessment to identify the threshold value of the CALLY index by adjusting the trial breakpoints within a predetermined range and selecting the point that maximized the model likelihood. Furthermore, a likelihood ratio test compared linear regression models, and bootstrapping resampling calculated 95% confidence intervals (95% CI) for inflection points [34].

Moreover, Kaplan‐Meier (KM) curves [35] were generated to compare survival probabilities among patients with depression, stratified according to different CALLY index thresholds. To assess the consistency of our findings, we performed independent sensitivity analyses. The first set involved conducting interaction tests for each covariate, aiming to identify any factors that could impact the results. These interaction tests were exploratory in nature and should be interpreted with caution. These additional analyses were crucial in validating the robustness of the results obtained.

In addressing missing data, missing values for continuous variables exceeding 1% were imputed with the mean value. For categorical variables, interpolated dummy variables were utilized. All statistical tests were two‐sided, and a *p*‐value < 0.05 was considered statistically significant. Statistical analyses were performed using R software (version 4.4.2; R Foundation for Statistical Computing, Vienna, Austria) and EmpowerStats software (version 4.2; Boston, Massachusetts, USA; https://www.empowerstats.com).

## 3 Results

### 3.1 Initial Participant Profile

This research encompassed four survey cycles (2005–2010 and 2015–2016). Following the exclusion of cases with incomplete information, the final analytic sample comprised 4,426 individuals.

Over a median follow‐up of 121 months, 657 individuals passed away, resulting in an overall all‐cause mortality rate of 14.84%. Table 1 outlines the initial demographic and clinical characteristics of the study population, categorized by survival status. Notable distinctions emerged between survivors and non‐survivors in almost all examined demographic and clinical parameters.

**Table 1:** Initial participant profile.^a^

Characteristics	Total	Alive	Deceased	*p*‐value
*N* (%)	4426	3769 (85.16)	657 (14.84)	
Sex, *n* (%)				< 0.001
Male	1822 (41.17)	1483 (39.35)	339 (51.60)	
Female	2604 (58.83)	2286 (60.65)	318 (48.40)	
Age, Mean ± SD	49.39 ± 17.24	46.27 ± 15.95	67.27 ± 12.98	< 0.001
Race, *n* (%)				< 0.001
Mexican American	812 (18.35)	752 (19.95)	60 (9.13)	
Other Hispanic	501 (11.32)	462 (12.26)	39 (5.94)	
Non‐Hispanic White	2016 (45.55)	1595 (42.32)	421 (64.08)	
Non‐Hispanic Black	843 (19.05)	727 (19.29)	116 (17.66)	
Other Race	254 (5.74)	233 (6.18)	21 (3.20)	
Education level, *n* (%)				< 0.001
Less than high school	653 (14.77)	529 (14.05)	124 (18.93)	
High school diploma	1927 (43.60)	1609 (42.74)	318 (48.55)	
More than high school	1840 (41.63)	1627 (43.21)	213 (32.52)	
Marital status, *n* (%)				< 0.001
Marriage/Living with partner	2320 (52.43)	2020 (53.61)	300 (45.66)	
Widowed/Divorced/Separated	1264 (28.56)	961 (25.50)	303 (46.12)	
Never married	841 (19.01)	787 (20.89)	54 (8.22)	
PIR, *n* (%)				< 0.001
< 1.3	1699 (38.39)	1428 (37.89)	271 (41.25)	
1.3–3.49	1282 (28.97)	1596 (42.35)	309 (47.03)	
> = 3.5	2040 (46.09)	745 (19.77)	77 (11.72)	
Hypertension, *n* (%)				< 0.001
No	3958 (89.59)	2331 (61.95)	242 (36.95)	
Yes	1845 (41.76)	1432 (38.05)	413 (63.05)	
Diabetes, *n* (%)				< 0.001
No	3671 (83.05)	3216 (85.42)	455 (69.47)	
Yes	749 (16.95)	549 (14.58)	200 (30.53)	
CRD, *n* (%)				< 0.001
No	3271 (73.90)	2843 (75.43)	428 (65.14)	
Yes	1155 (26.1)	926 (24.57)	229 (34.86)	
CVDs, *n* (%)				< 0.001
No	3733 (84.36)	3346 (88.80)	387 (58.90)	
Yes	692 (15.64)	422 (11.20)	270 (41.10)	
Malignancy, *n* (%)				< 0.001
No	3958 (89.59)	3456 (91.89)	502 (76.41)	
Yes	460 (10.41)	305 (8.11)	155 (23.59)	
Smoking, *n* (%)				< 0.001
No	2001 (45.25)	1788 (47.48)	213 (32.47)	
Yes	2421 (54.75)	1978 (52.52)	443 (67.53)	
Alcohol, *n* (%)				< 0.001
No	1290 (29.21)	1060 (28.18)	230 (35.06)	
Yes	3127 (70.79)	2701 (71.82)	426 (64.94)	
Moderate recreational activities, *n* (%)				< 0.001
No	2963 (66.98)	2451 (65.07)	512 (77.93)	
Yes	1461 (33.02)	1316 (34.93)	145 (22.07)	
BMI (kg/m^2^), Mean ± SD	30.31 ± 7.57	30.47 ± 7.62	29.44 ± 7.17	0.001
Albumin (g/dL), Mean ± SD	4.19 ± 0.35	4.22 ± 0.33	4.03 ± 0.39	< 0.001
Lymphocyte (10^3^/uL), Median (IQR)	2.10 (1.70, 2.60)	2.20 (1.80, 2.70)	1.90 (1.50, 2.40)	< 0.001
CRP (mg/dL), Median (IQR)	0.25 (0.10, 0.59)	0.24 (0.09, 0.55)	0.36 (0.13, 0.82)	< 0.001
Log‐CALLY index, Mean ± SD	4.56 ± 0.59	4.60 ± 0.58	4.33 ± 0.62	< 0.001
Follow‐up‐Time (month), Median (IQR)	121.0 (55.0, 145.0)	126.0 (57.0, 148.0)	70.0 (33.0, 105.0)	< 0.001

a Results in table:Normally distributed data were summarized as mean ± standard deviation, while skewed distributions were reported as median with interquartile range (IQR). For continuous variables, statistical significance was assessed with the Kruskal‐Wallis test. Categorical data are expressed as frequencies with percentages, and group differences were analyzed using Fisher's exact test.

The deceased group, on average, was much older than the surviving group (67.27 ± 12.98 years vs. 46.27 ± 15.95 years, *p* < 0.001). Additionally, The proportion of male participants was significantly elevated in the non‐survivor group compared to survivors (51.60% *vs.* 39.35%, *p* < 0.001). Sociodemographic factors also played a role in distinguishing the two groups, with a larger portion of non‐Hispanic whites in the deceased group (64.08% vs. 42.32%), a lower level of education (only 32.52% had completed high school or higher compared to 43.21% in the surviving group), and a significantly elevated prevalence was also observed among those not in a marital partnership (46.12% vs. 25.50%, *p* < 0.001). From an economic perspective, the deceased group demonstrated a substantially greater proportion of subjects within the intermediate poverty‐income bracket (1.3–3.49) relative to survivors (47.03% *vs.* 42.35%, *p* < 0.001).

Regarding clinical characteristics and comorbidities, the deceased group had a significantly heavier disease burden, with higher prevalence rates of hypertension, diabetes, chronic kidney disease, cardiovascular diseases, and malignancies (*p* < 0.001). Lifestyle factors also differed significantly, as the deceased group demonstrated a higher smoking rate and lower rates of alcohol consumption and moderate engagement in recreational activities (*p* < 0.001).

Additionally, the deceased individuals exhibited lower serum albumin and lymphocyte levels, along with higher CRP levels. The median CALLY index (IQR) was 4.56 (IQR: 3.97–5.15), with the deceased group showing a significantly lower CALLY index compared to the surviving group (4.33 ± 0.62 vs. 4.60 ± 0.58, *p* < 0.001).

### 3.2 Relationship of the Log‐CALLY Index With Overall Mortality in Depressed Individuals

After applying logarithmic transformation to the CALLY index values, we identified potential confounders based on both clinical relevance and statistical significance (*p* < 0.05) in univariate analyses, in accordance with the method described in the Statistical Analysis section. This led us to construct four adjusted models for our analysis. The initial crude model was left unadjusted, serving as a baseline for comparison. The initial regression model (Model 1) incorporated fundamental demographic characteristics including gender, age, ethnicity, educational attainment, marital status, and poverty‐income ratio. Subsequently, Model 2 expanded the analytical framework by incorporating additional covariates related to health status, specifically accounting for body mass index, diabetes mellitus, high blood pressure, tobacco use, and alcohol intake. Finally, Model 3 included additional adjustments for lifestyle factors and various chronic diseases, such as moderate leisure activities, malignancies, CVDs and CRD, building upon the foundation of Models 1 and 2.

As detailed in Table 2, Cox proportional hazards models were employed to assess the association. When analyzed as a continuous measure, the log‐transformed CALLY index showed a hazard ratio of 0.48 (95% CI: 0.43–0.55) in the unadjusted analysis. Following multivariable adjustment in Model 3, the HR was 0.59 (95% CI: 0.51–0.68). The results indicate that each one‐unit increment in the log‐transformed CALLY index corresponds to a 41% reduction in all‐cause mortality risk in individuals with depression.

**Table 2:** Univariate and multivariate results by cox regression.

Characteristic	Crude model^a^		Model 1^b^		Model 2^c^		Model 3^d^	
	HR (95%CI)	*p* value	HR (95%CI)	*p* value	HR (95%CI)	*p* value	HR (95%CI)	*p* value
Log‐CALLY index	0.48 (0.43, 0.55)	< 0.0001	0.60 (0.52, 0.68)	< 0.0001	0.57 (0.50, 0.66)	< 0.0001	0.59 (0.51, 0.68)	< 0.0001
Index quartile								
Quartile 1	Ref		Ref		Ref		Ref	
Quartile 2	0.75 (0.62, 0.91)	0.0037	0.72 (0.59, 0.87)	0.0010	0.72 (0.59, 0.87)	0.0007	0.75 (0.62, 0.91)	0.0041
Quartile 3	0.46 (0.37, 0.57)	< 0.0001	0.48 (0.39, 0.60)	< 0.0001	0.49 (0.39, 0.62)	< 0.0001	0.52 (0.41, 0.65)	< 0.0001
Quartile 4	0.41 (0.32, 0.51)	< 0.0001	0.59 (0.47, 0.74)	< 0.0001	0.54 (0.43, 0.69)	< 0.0001	0.60 (0.47, 0.76)	< 0.0001
*p* for trend*		< 0.0001		< 0.0001		< 0.0001		< 0.0001

*To assess linear trends, categorical variables were analyzed as if they were continuous parameters.

^a^ Crude Model: Unadjusted for covariates.

^b^ Model 1: Adjusted for gender, race, age, marital status, education level and PIR.

^c^ Model 2: Building upon Model 1, further adjustments were made for BMI, diabetes, hypertension, smoking and alcohol.

^d^ Model 3: Expanding on Model 2, additional adjustments were made for moderate recreational activities, malignancies, CVDs and CRD.

To mitigate potential biases in the overall sample analysis, the log‐CALLY index was grouped into four quartiles, where Q1 (the lowest range) was established as the baseline for comparison.

Through sensitivity analysis, we found that patients in the highest quartile (Q4) consistently showed lower hazard ratios (HR) compared to those in Q1 across all models. The corresponding hazard ratios across progressively adjusted models were as follows: crude model: 0.41 (95% CI 0.32–0.51); Model 1: 0.59 (95% CI 0.47–0.74); Model 2: 0.54 (95% CI 0.43–0.69); and Model 3:0.60 (95% CI 0.47–0.76). To evaluate trend linearity, categorical variables were handled as continuous numeric variables. All models displayed a significant linear trend (*p*‐trend < 0.001). In summary, Analyses of both continuous and categorical forms of the CALLY index reveal a robust inverse relationship with mortality from all causes.

**Figure 2:**
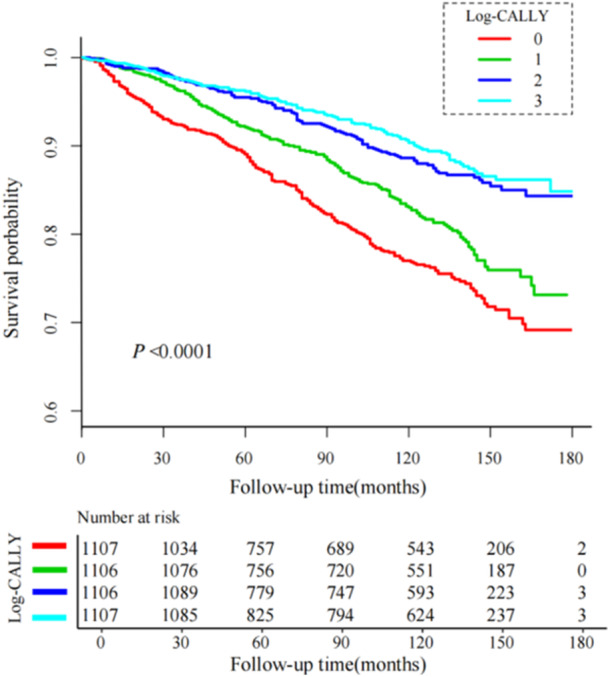
Kaplan‐Meier survival curves stratified by quartiles of the log‐transformed CALLY index among individuals with depression.

As shown in Figure 2, Kaplan‐Meier analysis revealed marked differences in survival outcomes when participants were stratified by CALLY index levels, with statistically significant separation between groups (*p* < 0.001). Participants with lower CALLY index measurements exhibited significantly reduced survival probabilities relative to those with elevated levels. Furthermore, a consistent inverse relationship was observed, whereby ascending CALLY index values corresponded with progressively lower all‐cause mortality rates.

### 3.3 Identification of Nonlinear Relationships

**Figure 3:**
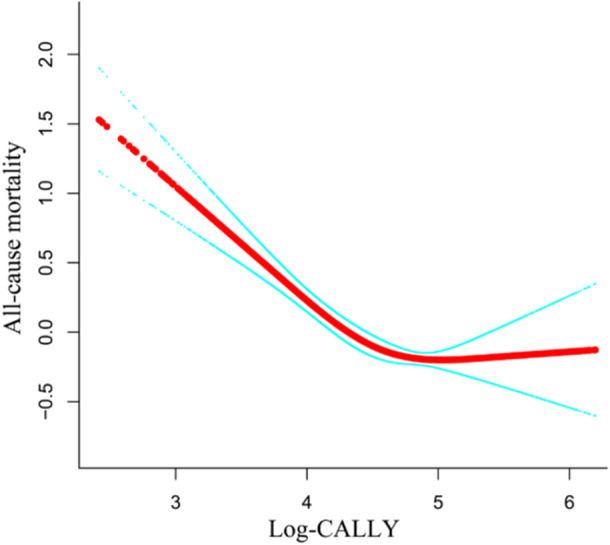
Restricted Cubic Spline Analysis. *Note:* The red curve represents the smoothed association based on restricted cubic spline analysis, with the blue band indicating the corresponding 95% confidence interval.

In our study, the relationship of the CALLY index with mortality from all causes among individuals with depressive disorders was assessed using RCS analysis. The results indicated a nonlinear l‐shaped negative correlation (*p* < 0.001) (Figure 3), with the nonlinear association showing a transition point at 4.09 on the Log‐CALLY index scale (Table 3). When the Log‐CALLY index was less than 4.09, the all‐cause mortality hazard ratio (HR) was 0.39 (95% CI: 0.29–0.53, *p* < 0.0001), this corresponded to an approximate 61% decrease in all‐cause mortality risk with each one‐unit increment in the index value, demonstrating a protective effect. When the Log‐CALLY index exceeded 4.09, the mortality HR was 0.76 (95% CI: 0.61–0.94, *p* = 0.0102). Although still statistically significant, the magnitude of protection substantially diminished beyond this threshold, with every unit rise in the index corresponding to merely an approximate 24% reduction in all‐cause mortality (Table 3).

**Table 3:** Analyzing threshold effects.

Models	All‐cause mortality	
	HR (95%CI)	*p* value
Mode I		
One line effect	0.59 (0.51, 0.68)	< 0.0001
Model II		
Turning point (K)	4.09	
CALLY index < K	0.39 (0.29, 0.53)	< 0.0001
CALLY index ≥ K	0.76 (0.61, 0.94)	0.0102
*p* value for LRT test*	0.004	
95% CI for turning point	3.85, 4.26	

*Note:* Results are displayed as hazard ratio (HR) (95% confidence intervals) accompanied by *p*‐values. Model I assumes a linear association, whereas Model II incorporates a nonlinear relationship. The likelihood ratio test was employed to compare the two models with.

* *p*< 0.05 denoting a statistically significant difference in Model II over Model I.

### 3.4 Subgroups Analysis

**Figure 4:**
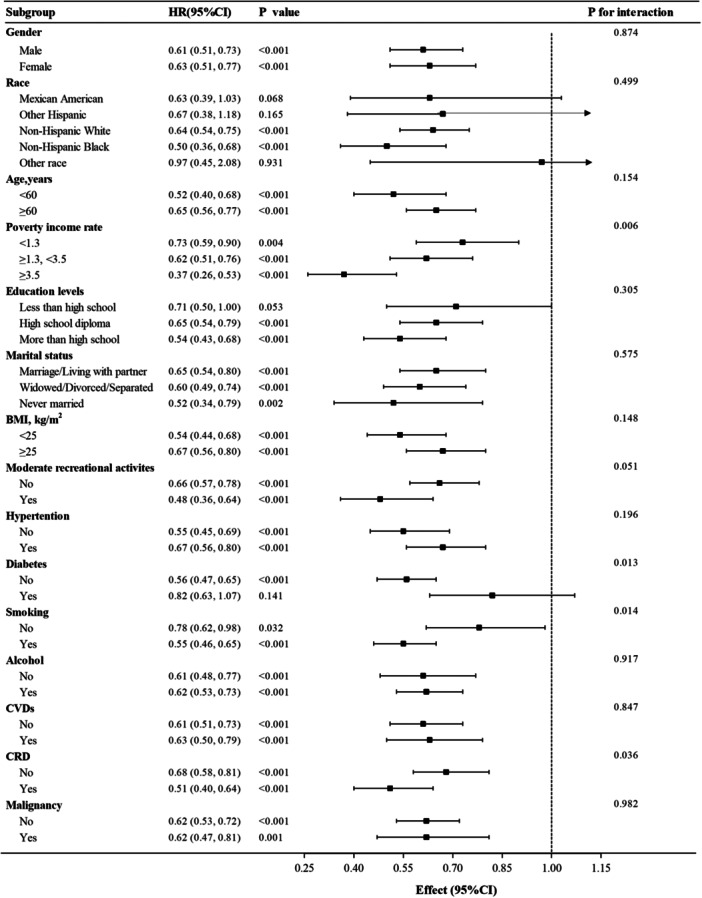
Subgroup Analysis.

We conducted a subgroup analysis stratified by sex, age, race, education level, marital status, PIR, BMI, diabetes, hypertension, smoking, alcohol consumption, moderate leisure activities, malignancies, cardiovascular diseases, and chronic respiratory diseases. Multiple analytical methods consistently demonstrated that depressed patients with lower CALLY index values faced substantially elevated mortality risk from all causes (*p* < 0.05), as illustrated in Figure 4. Particularly noteworthy were the statistically significant interactions observed between PIR, diabetes, smoking, and CRD (interaction *p*‐values of 0.006, 0.013, 0.014, and 0.036, respectively). Conversely, no significant interactions were detected among other covariate subgroups (*p* > 0.05). These subgroup analyses were exploratory in nature and should be interpreted with caution.

### 3.5 Supplementary Analyses

Supplementary analyses were conducted to address methodological considerations regarding study population and outcome definition. Stratified analyses by PHQ‐9 depression severity demonstrated consistent inverse associations between the CALLY index and all‐cause mortality in participants with mild (scores 5–9: HR 0.61, 95% CI 0.51–0.72) and moderate‐to‐severe symptoms (scores ≥ 10: HR 0.59, 95% CI 0.46–0.74), with no significant interaction (P‐interaction = 0.829; Supporting Table S1). Restricted cubic spline analysis in the moderate‐to‐severe subgroup showed a nonlinear dose‐response relationship comparable to the primary cohort (Supporting Figure S1).

For cardiovascular mortality (167 events), continuous modeling revealed a significant inverse association (HR 0.68, 95% CI 0.52–0.89, *p* = 0.005), while quartile‐specific estimates were protective (HRs 0.62–0.68 across quartiles 2–4 vs. quartile 1) but the trend test was non‐significant (*p* for trend = 0.646; Supporting Table S2).

## 4 Discussion

Drawing from an extensive retrospective cohort, this investigation comprised 4,426 adults with clinically confirmed depression, results showed that higher levels of CALLY were linked to decreased risks of mortality, showcasing an l‐shaped negative correlation. Comprehensive subgroup evaluations and interaction assessments consistently corroborated the robustness of our results. The supplementary analyses confirmed that the associations between the CALLY index and all‐cause mortality remained consistent across PHQ‐9 severity strata, and that the nonlinear patterns in the moderate‐to‐severe subgroup were comparable to those in the primary cohort (Supporting Table S1 and Figure S1). The protective effect also extended to cardiovascular mortality in continuous analysis (Supporting Table S2), although the non‐significant quartile trend is likely due to limited statistical power from only 167 events.

Substantial evidence now connects immunological dysregulation, chronic inflammation, and nutritional deficiencies to depression pathogenesis, with particular vulnerability observed in elderly populations and chronically ill patients [36, 37]. Issues like malnutrition, chronic inflammation, and autoimmune disorders can exacerbate depressive symptoms, leading to a lack of volitional activity among patients, and in severe cases, resulting in suicidal and self‐harming behaviors. These detrimental outcomes can significantly impact both the longevity and quality of life of those suffering from depression [38]. As a result, healthcare professionals are now exploring various indicators, such as immune, nutritional, and inflammatory markers, which are increasingly being regarded as potential biomarker candidates for predicting the prognosis of depression.

Our research, with a larger sample size, confirms previous findings and further utilizes the NHANES database while investigating the connection linking CALLY index levels to overall mortality risk in adults diagnosed with depression. Derived from three key parameters—lymphocyte count, serum albumin, and CRP—the CALLY index acts as an effective measure for assessing nutritional status, immune performance, and inflammatory responses [17, 18]. Currently, The CALLY index possesses significant predictive utility for adverse outcomes, especially in assessing prognosis related to respiratory diseases, cardiovascular conditions, and malignancies [39, 40, 41].

Early malnutrition can exacerbate depression in later life [42]. With an extended biological half‐life, serum albumin serves as an essential plasma protein routinely employed to assess nutritional condition and protein synthesis capacity. Unfortunately, malnutrition and inflammation in patients with different diseases can impede the production of serum albumin [43].

Previous research has suggested that there is a link between low serum albumin levels and depression [44, 45]. Tryptophan, a key neurotransmitter involved in mood regulation, is significantly influenced by albumin's role in transporting and metabolizing tryptophan. Low levels of albumin can inhibit the availability of tryptophan, thereby impairing serotonin synthesis and exacerbating depressive symptoms [46]. Moreover, this process activates inflammatory responses that release cytokines, which in turn enhance the activity of the HPA axis. This leads to an increase in stress hormone levels such as ACTH and cortisol, ultimately promoting the development of depression [47].

Evidence from prior investigations indicates that serum albumin levels are inversely related to depressive symptom severity in individuals diagnosed with HIV infection [48]. A study involving US adults demonstrated that the odds ratio decreased linearly with each quartile increase in albumin levels (*p* for trend = 0.001), indicating a potential protective effect against depressive symptoms [49]. Additionally, research by Ding indicated that the PNI, which is based on albumin levels, has been shown to confer protection against overall and cardiovascular death [50].

As modern neuroimmunology advances, the connection between the immune system and depression is gaining recognition. It is now understood that medication alone is not always sufficient to treat all symptoms of depression. The body's immune regulatory functions serve a critical function in maintaining emotional stability and facilitating long‐term recovery. This highlights the importance of considering the immune system in the treatment and management of depression.

Lymphocytes are crucial for adaptive immunity and play a role in regulating emotions and contributing to the development of depression. Through the secretion of cytokines, modulation of neuroinflammation, and impact on neuroplasticity and synaptic function, lymphocytes influence the physiological and psychological aspects of mental health. Lymphocyte counts play a vital role in evaluating immune function in various settings, given their sensitivity to numerous factors. When combined with other inflammatory and immune biomarkers like the NLR, PLR, and MLR, lymphocyte counts become even more valuable for assessing overall immune health.

Cheng et al [51] conducted a review involving 2,264 individuals with depression and found that the NLR, PLR, and MLR were significantly higher in the depressive cohort than in the healthy controls. What's more, in a cross‐sectional study, Shan et al [52] discovered a nonlinear correlation between PLR and both depression and symptom severity within a population of US adults. Furthermore, Amitai et al [53] identified that elevated NLR and PLR values in children and adolescents with depression may be more closely associated with suicidal behaviors.

The impact of inflammation on depression has been extensively explored; however, the exact ways in which inflammation contributes to the development of depression are still not fully understood. Studies have shown that individuals with a pro‐inflammatory state are more likely to experience depression [54], with those at higher risk demonstrating heightened inflammatory responses compared to those at lower risk [55]. Regulation of CRP transcription involves cytokines and transcription factors, with IL‐6 being a key stimulator for hepatocytes to produce CRP. The plasma concentration of IL‐6 significantly and rapidly increases during inflammation, infection, or tissue injury. Similarly, CRP is classic biomarkers that reflect a relatively stable state of systemic chronic inflammation [55].

A pooled analysis of available research indicates that people experiencing depression often exhibit elevated inflammatory markers such as CRP and IL‐12, indicating a more consistent inflammatory phenotype [56]. Osimo et al [57] found that one‐quarter of individuals with depression display low grade inflammation, with over half showing mildly increased CRP levels. Recently, a study conducted in a population of 9,759 individuals from northeastern Iran revealed a positive correlation between depression and elevated high‐sensitivity CRP (hs‐CRP) levels [58], particularly among males. Additionally, Nguyen et al [59] further identified that increased C‐reactive protein concentrations among patients with comorbid depression and diabetes are associated with elevated mortality risk.

This study, utilizing the NHANES database, intended to explore how the CALLY index relates to overall mortality risk in individuals diagnosed with depression, using data from a large national cohort. We meticulously adjusted for various demographic and other influencing factors to ensure the credibility and applicability of our results. We verified the stability of our conclusions through various sensitivity tests and stratified analyses. While our study offers several advantages, it is not without limitations. Due to the retrospective design of the study, we were unable to assess longitudinal changes in the relationship between the CALLY index and mortality outcomes, thereby limiting our scope to establish causal relationships. Additionally, the reliance on the PHQ‐9 scale for diagnosing depression introduced potential subjectivity that may have affected the accuracy of our results. Although we adjusted for multiple covariates, the potential impact of unmeasured confounders on our results cannot be entirely excluded. Future studies ought to examine the potential causative pathways suggested by our results and resolve the methodological shortcomings described herein. However, caution is warranted when extrapolating to other populations or healthcare systems.

## 5 Conclusion

Our findings demonstrate that diminished CALLY index measurements correlate strongly with depression susceptibility and serve as indicators for heightened mortality risk among those with the condition. Analysis using RCS demonstrated an L‐shaped association between the Log‐CALLY index and mortality, identifying 4.09 as the threshold value. When the Log‐CALLY value falls below this threshold, it should serve as a crucial indicator for intensified multidisciplinary care, including nutritional interventions like protein supplementation, infection prevention measures, and monitoring of inflammatory markers. This relationship is especially pronounced among middle‐ and low‐income populations, smokers, and individuals with diabetes or chronic respiratory conditions. Therefore, a comprehensive assessment of nutritional status, inflammation levels, and immune function is vital in managing patients with depression to identify those at a heightened risk of mortality early on. By systematically targeting these modifiable factors, mental health professionals can achieve better patient prognoses and refine therapeutic approaches for depressive disorders.

## Author Contributions

**Yin Zhang:** data curation, writing – original draft, writing – review and editing, software, conceptualization, methodology. **Rula Sa:** formal analysis, validation, investigation. **Ruiting Ma:** conceptualization, methodology, software, data curation.

## Ethics Statement

This study utilized publicly available data from the National Health and Nutrition Examination Survey (NHANES), which was approved by the National Center for Health Statistics (NCHS) Ethics Review Board (https://www.cdc.gov/nchs/nhanes/about/erb.html#print). All participants provided informed consent at the time of data collection by NHANES. As this study utilized publicly available data from the NHANES database, no additional ethical approval or consents were required.

## Conflicts of Interest

The authors declare no conflicts of interest.

## Transparency Statement

The lead author Ruiting Ma, Lijun Tong affirms that this manuscript is an honest, accurate, and transparent account of the study being reported; that no important aspects of the study have been omitted; and that any discrepancies from the study as planned (and, if relevant, registered) have been explained.

## Supporting information


**Supporting File:** hsr272387‐sup‐0001‐Supplementary_Material.docx.

## Data Availability

The datasets supporting the conclusions of this article are available in the National Health and Nutrition Examination Survey (NHANES) repository. These data are publicly accessible and can be found at: https://www.cdc.gov/nchs/nhanes/.
