# Supplementary material for: The Impact of the CALLY Index on All‐Cause Mortality in Patients With Depression: A Longitudinal Analysis Using NHANES Data
Source: Health Sci Rep. 2026 Apr 19;9(4):e72387. doi: 10.1002/hsr2.72387 (PMC13092223; doi:10.1002/hsr2.72387)
Supplement: Supplementary file 1 — Supporting File: hsr272387‐sup‐0001‐Supplementary_Material.docx. [file HSR2-9-e72387-s001.docx]

****Supplementary Table 1. Stratified analyses and test for interaction by depression severity****

| **Characteristic** | ****Mild symptoms^a^**** | | ****Moderate-to-severe symptoms^b^**** | | ***P* **for** interaction** |
| --- | --- | --- | --- | --- | --- |
|  | HR(95%CI) | *P* value | HR(95%CI) | *P* value |  |
| **Crude Model^c^** | 0.46(0.39,0.54) | <0.0001 | 0.52(0.42,0.65) | <0.0001 | 0.384 |
| **Model 1^d^** | 0.61(0.51,0.72) | <0.0001 | 0.59(0.46,0.74) | <0.0001 | 0.829 |

^a^**Mild symptoms** defined as PHQ-9 scores 5-9.

^b^**Moderate-to-severe symptoms** defined as PHQ-9 scores ≥10.

^c^Crude Model: Unadjusted for covariates.

^d^Model 1: Adjusted for gender, race, age, marital status, education level, PIR, BMI, diabetes, hypertension, smoking, alcohol, moderate recreational activities, malignancies, CVDs and CRD.

| 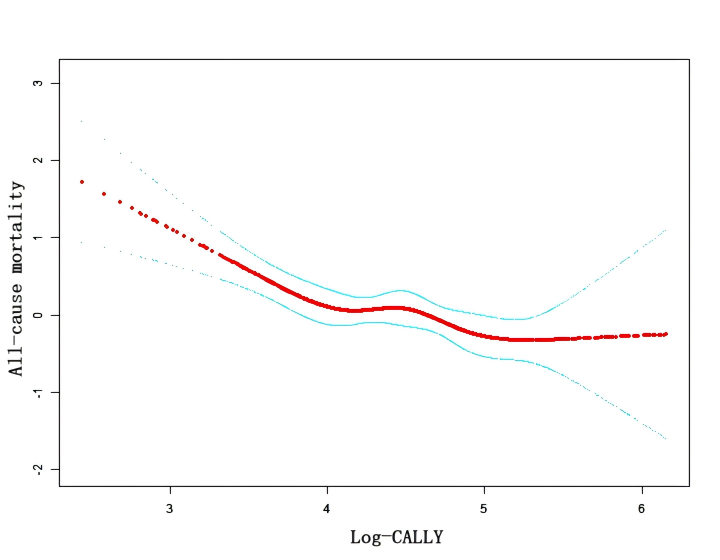 |
| --- |
| **Supplementary Figure 1. Association between Log-CALLY index and All-cause mortality: results from Restricted Cubic Spline Analysis**  Note: Moderate-to-severe depressive symptoms were defined as PHQ-9 scores ≥10.  The red curve represents the smoothed association based on restricted cubic spline analysis, with the blue band indicating the corresponding 95% confidence interval. |

**Supplementary Table 2. Association between CALLY index and Cardiovascular mortality: results from Cox regression analyses**

|  | **Crude Model^a^** | | **Model 1^b^** | | **Model 2^c^** | | **Model 3^d^** | |
| --- | --- | --- | --- | --- | --- | --- | --- | --- |
|  | **HR(95%CI)** | ***P* value** | **HR(95%CI)** | ***P* value** | **HR(95%CI)** | ***P* value** | **HR(95%CI)** | ***P* value** |
| Log-CALLY index | 0.64 (0.49, 0.83) | 0.0008 | 0.65 (0.50, 0.85) | 0.0015 | 0.64 (0.49, 0.84) | 0.0013 | 0.68 (0.52, 0.89) | 0.0052 |
| Index quartile |  |  |  |  |  |  |  |  |
| Quartile 1 | Ref |  | Ref |  | Ref |  | Ref |  |
| Quartile 2 | 0.69 (0.47, 1.00) | 0.0497 | 0.75 (0.51, 1.09) | 0.1322 | 0.72 (0.49, 1.06) | 0.0986 | 0.78 (0.53, 1.16) | 0.2205 |
| Quartile 3 | 0.66 (0.42, 1.03) | 0.0658 | 0.61 (0.38, 0.96) | 0.0328 | 0.61 (0.38, 0.97) | 0.0371 | 0.68 (0.42, 1.09) | 0.1092 |
| Quartile 4 | 0.61 (0.38, 0.96) | 0.0348 | 0.62 (0.39, 1.00) | 0.0489 | 0.60 (0.37, 0.98) | 0.0424 | 0.62 (0.38, 1.03) | 0.0644 |
| P for trend***** |  | 0.6853 |  | 0.6499 |  | 0.7262 |  | 0.9437 |

**^a^**Crude Model: Unadjusted for covariates.

**^b^**Model 1: Adjusted for gender, race, age,marital status, education level and PIR.

**^c^**Model 2: Building upon Model 1, further adjustments were made for BMI, diabetes, hypertension,smoking and alcohol.

**^d^**Model 3: Expanding on Model 2, additional adjustments were made for moderate recreational activities, malignancies,CVDs and CRD.

**^*^**To assess linear trends, categorical variables were analyzed as if they were continuous parameters.
